# Long-Term Halo Follow-Up Confirms Less Invasive Treatment of Low-Grade Cartilaginous Tumors with Radiofrequency Ablation to Be Safe and Effective

**DOI:** 10.3390/jcm10091817

**Published:** 2021-04-22

**Authors:** Hendricus Nijland, Jelle Overbosch, Joris J. W. Ploegmakers, Thomas C. Kwee, Paul C. Jutte

**Affiliations:** 1 Department of Orthopaedic Surgery, University Medical Center Groningen, 9713GZ Groningen, The Netherlands; j.j.w.ploegmakers@umcg.nl (J.J.W.P.); p.c.jutte@umcg.nl (P.C.J.); 2 Department of Radiology, University Medical Center Groningen, 9713GZ Groningen, The Netherlands; j.overbosch@umcg.nl (J.O.); t.c.kwee@umcg.nl (T.C.K.)

**Keywords:** percutaneous, ablation, low-grade cartilaginous tumor, halo, results, complications

## Abstract

Background: Radiofrequency ablation (RFA) is a minimally invasive alternative in the treatment of bone tumors. Long-term follow-up has not been described in current literature. Detailed analysis of mid- and long-term follow-up after RFA treatment for a cohort of patients with low-grade cartilaginous tumors (atypical cartilaginous tumors and enchondroma) was performed. The results, complications, and development of halo dimensions over time are presented. Methods: Data of all patients with an RFA procedure for an ACT between 2007–2018 were included. Ablation area is visible on baseline MRI, 3 months post-procedure, and is called halo. Volume was measured on MR images and compared to different follow-up moments to determine the effect of time on halo volume. Follow-up was carried out 3 months and 1, 2, 5, and 7 years after the procedure. Occurrence of complications and recurrences were assessed. Results: Of the 137 patients included, 82 were analyzed. Mean follow-up time was 43.6 months. Ablation was complete in 73 cases (89.0%). One late complication occurred, while no recurrences were seen. Halo dimensions of height, width, and depth decreased with a similar rate, 21.5% on average in the first year. Subsequently, this decrease in halo size continues gradually during follow-up, indicating bone revitalization. Conclusion: RFA is a safe and effective treatment in low-grade cartilaginous tumors with an initial success rate of 89.0%. Extended follow-up shows no local recurrences and gradual substitution of the halo with normal bone.

## 1. Introduction

Chondrosarcoma are the most common primary bone tumors in adults [1]. Atypical cartilaginous tumors (ACT), formerly known as chondrosarcoma grade 1, are the most common type of chondrosarcoma. An ACT is a cartilage-forming tumor with primary location in the long bones (mainly femur) [2]. The five-year survival rate reported in literature is 93% [2]. An ACT can show aggressive local growth with (very) low tendency to metastasize. Diagnosis is regularly made coincidently from MR or CT imaging for common skeletal symptoms [2,3]. Differentiation between ACT and enchondroma is not always clear on an MRI or after biopsy given the continuum between these diagnoses.

The first choice of treatment has been topic of discussion in recent literature. Since ACTs show resistance to both radiotherapy and chemotherapy, treatment is often either surgical or conservative, with frequent follow-up [4]. In surgical treatment, the most common options are considered intralesional curettage and resection, with a tendency towards intralesional curettage [3,5]. Success rates (i.e., no residue or recurrence) are about 90% for curettage and 95% for resection, with complications occurring in 2.8% and 13.3% of cases, respectively [3,6]. The use of radiofrequency ablation (RFA) for ACT as a minimally invasive alternative to the above-mentioned options has been developed since 2007. Out of 189 consecutive patients treated with RFA, the success rate was 84.4%, with a complication in 7.9% of patients [7]. Therefore, RFA is considered effective at achieving local tumor control in ACT of long bones. However, long-term follow-up was not described. 

In RFA, a small tract is drilled towards the tumor under image guidance (CT, fluoroscopy, or computer assisted surgery). Through this, tract a needle is brought up and tumor destruction is achieved by the application of local heat for several minutes [8,9]. Cell death is achieved by desiccation and instantaneous protein coagulation at temperatures over 60 °C. Bone tissue is sensitive to heating at temperatures over 47 °C. At temperatures between 50–60 °C, it takes 1 to 6 minutes for necrosis to occur [10,11,12]. The temperature at the tip of the needle, normally between 75–90 °C, is different than the temperature that reaches the edge of the ablation halo because of heat loss during distribution. A special point of interest is the outer layer of the ablation zone. Tissue that does not reach complete necrosis might show remodeling over time. We hypothesized a remodeling process to occur, similar to the remodeling after a fracture. From literature, it is known that in the first weeks after a fracture, osteoclasts remove the necrotic tissue. Subsequently, osteoblast activity leads to calcification and the formation of a new trabecular bone [13]. 

Less invasive treatment leads to better functional outcome, lower hospitalization periods, fewer complications, and, in general, higher patient satisfaction [14,15]. Therefore, RFA might lead to further improvement of outcome and satisfaction. Literature on RFA for ACT has so far been limited [5,7,16]. Therefore, the evolution of ablated tissue over time and recurrence rates after ablation are unknown. The present paper aims to evaluate (mid/long-term) follow-up with regular MRI scans and an analysis of the ablated tissue over time.

## 2. Materials & Methods

### 2.1. Procedure

Diagnosis was based on (incidental) conventional radiographic findings and confirmed on additional MRI. Given the possibility of metastatic disease after ACT and majority of symptoms (e.g., pain, uncertainty), the decision was made to also include cases in which differentiation was unclear.

RFA was generally carried out in the department of interventional radiology under CT-guidance. In case of large tumors, difficult localizations or comorbidities treatment were carried out in the operation room, image guided (fluoroscopy or computer assisted surgery) depending on tumor localization, volume, and comorbidities. Tumors with a diameter over 6 cm were generally treated in the operation room because of the possibility to place a prophylactic osteosynthesis to prevent fracture. Procedures were carried out under local anesthesia. Ablation was performed with a Cooltip^®^ RFA needle (Medtronics, Minneapolis, MN, USA).

**Figure 1:**
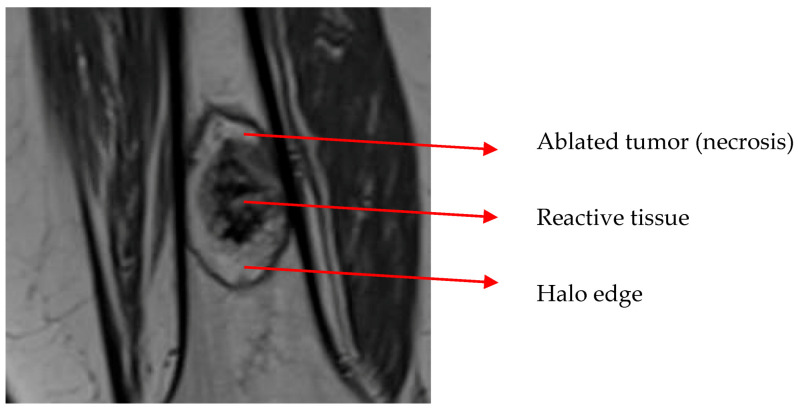
Ablation halo around the tumor. The dark outer area of the halo consists of reactive tissue (collateral bone ablation).

All follow-up was performed in the same center. The first follow-up was at 3 months. A baseline MRI (unenhanced T1-weighted, fat-suppressed T2-weighted, and gadolinium-enhanced sequences in two perpendicular planes with 4-mm slice thickness) was made to determine the exact ablation area. On an MRI, this area is depicted as an ellipse around the tumor tissue. This ablation area is called the ‘halo’. This elliptic halo consists of granulation tissue as a response to ablation (see Figure 1). Dierselhuis et al. found the amount of cell death to correlate well with the MRI aspect [14]. All measurements of halo volume were first performed by one blinded investigator. Different follow-up moments within the same patients were not measured consecutively to avoid bias. Subsequently, control measurements were performed by another investigator from the same institution, blinded from the initial measurement result. 

Complete ablation with a margin of >2 mm all around was considered R0. Complete ablations without this margin were called R1 and incomplete ablations R2. Follow-up was planned for 1, 2, 5, 7, and 10 years after the procedure [17]. 

### 2.2. Data Collection

Data were collected from a prospectively kept tumor database. This study was performed in accordance with the Declaration of Helsinki. According to regulations of the Medical Ethical Review Board of University Medical Center Groningen, patients were informed by means of written information about the fact that anonymous data of the procedure could be used for the evaluation of care and scientific research. As the procedure was part of usual care, no written or verbal consent was necessary (ethical approval number: METC UMCG 20140028).

All 183 patients with an ACT who were treated with RFA between 2007 and 2018 were considered for inclusion. Reason for exclusion was intralesional curettage following RFA. For the one-year follow-up moment, a difference of 4 months was accepted. For the later time intervals, the maximum accepted difference was 20%. Given the slow growth potential of ACT, tissue outside the ablation halo on the baseline MRI (3 months post-operative) has to be the result of incomplete ablation instead of recurrence. 

### 2.3. Data Analysis

**Figure 2:**
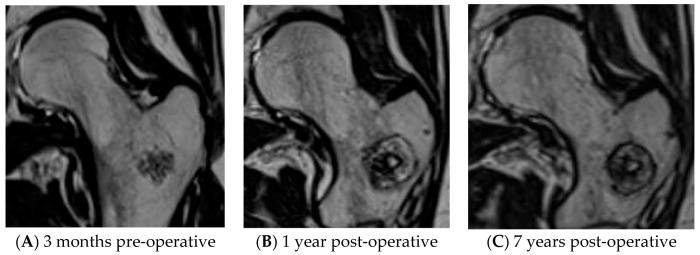
Low-grade cartilaginous tumor in femur before and after RFA treatment. (**A**) ACT on pre-operative MRI. (**B**) ACT on MRI, 1 year after RFA. (**C**) ACT on MRI, 7 years after RFA.

For all patients, tumor volume and halo volume at baseline and after 1, 2, 5, and (if applicable) 7 years was determined. From MR images, both tumor and halo height, width, and depth were measured. Elliptical volume was calculated according to the following formula: 1/6 × π × width × depth × height [7]. To evaluate halo volume, the decrease in volume over time was determined for the periods baseline–1 year, baseline–2 years, baseline–5 years, and baseline-7 years follow-up. Figure 2A–C depicts MR images of an ACT in the femur, the ablation halo at baseline and 7 years follow-up.

Data were analyzed using SPSS Statistics v25 (IBM, Armonk, NY, USA). Data were tested on normality of distribution. To test if the volume decrease over time was significant between the different intervals, linear regression was used. To examine the possible differences between the different time intervals, separate Wilcoxon signed rank tests were performed. For all tests, an alpha of 0.05 was chosen. 

## 3. Results

**Figure 3:**
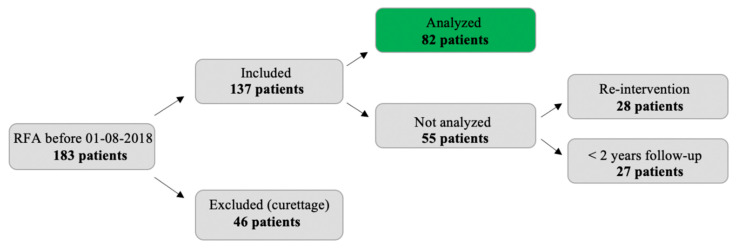
Flow chart indicating reasons for exclusion.

In 46 patients, intralesional curettage was performed following RFA (in 24 patients as part of a trial with standard curettage 4 months after RFA) [15]. These patients were excluded from analysis. The other 137 patients were eligible for analysis. For 28 patients, a re-intervention was carried out within a year (13 times for a complication, 12 times because of incomplete ablation, and 3 times for a different procedure in the same bone). Finally, 27 patients had less than 2 years follow-up for other reasons (non-adherence, death, other severe disease, follow-up at a different center, other imaging modality than MRI). The halos of the remaining 82 patients were analyzed (see Figure 3). 

In 73 out of 82 patients (89.0%), complete ablation (R0/R1) was achieved. In these 82 cases, one complication (a temporary radial nerve palsy) occurred and no recurrences were found after complete ablation. Halo volume over time was analyzed for all 82 patients. In 45 patients, total follow-up was 2 years. From the other 37 patients, 30 had 5 years follow-up, while seven patients had 7 years follow-up. Follow-up time was 43.8 months on average (range 19–100 months). Table 1 summarizes the general characteristics of the study population.

**Figure 4:**
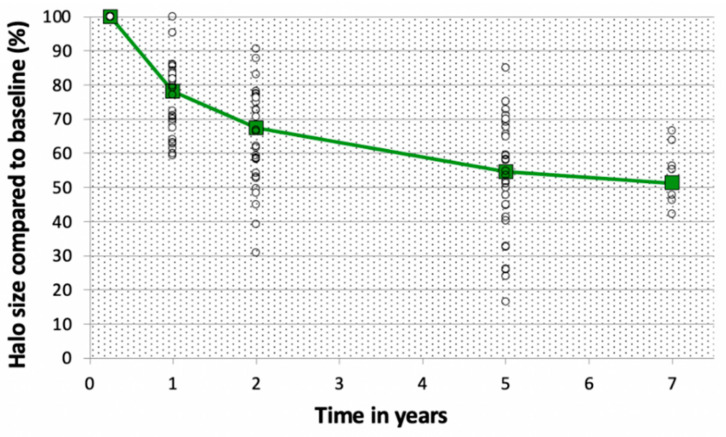
Halo size compared to baseline value (%) of the ablation halo relatively to the volume at baseline. Percentages are expressed relatively to the volume at baseline. White circles depict the individual cases, the green line depicts the average halo size compared to baseline.

Average halo volume at baseline (=28.6 cm^3^) was 3.7 times larger than the tumor volume (7.67 cm^3^ on average). Halo volume decreased over time (see Figure 4). This was the result of an equal decrease in height, width, and depth as depicted in Table 2. Volume decrease is expressed relatively to the average volume at baseline (with baseline value separately determined for only the samples with a measurement on that specific time interval). For cases with a measurement both at baseline and after 1 year, the average halo volume decreased from 28.6 to 22.5 cm^3^ (=−21.5%). Between 1 and 2 years, average volume decreased from 22.4 to 19.4 cm^3^ (=−13.5%). Between 2 and 5 years, this decrease was from 14.9 to 12.4 cm^3^ (=−5.56% annually), and from 5 to 7 years, 7.45 to 6.72 cm^3^ (=−4.90% annually). Since not all patients attended all follow-up moments, the absolute volume decrease is mentioned here (not relatively to the average volume at baseline). In none of the patients was an increase in halo size found.

Linear regression showed halo volume decrease over time (*p* < 0.001). Separate Wilcoxon signed ranks tests demonstrated a significant decrease in volume for every step in follow-up (baseline–1 year *p* < 0.001, 1 year–2 years *p* < 0.001, 2 years–5 years *p* < 0.001, 5 years–7 years *p* = 0.043).

## 4. Discussion

This is the first study to examine the results, complications, and halo development during extensive follow-up after RFA for low-grade cartilaginous tumors (ACT, enchondroma). A total of 82 patients with minimum 19 months follow-up was analyzed in the study. Ablation was successful in 73 of 82 cases, complications were rare, and halo volume gradually decreased over time. Given the fact additional treatment was carried out for patients with larger residues and for some of the complications, these numbers are slightly lower in clinical practice. 

On MRI, the ablation area is depicted as an elliptic halo around the tumor. This halo consists of granulation tissue as a response to ablation. Ablation was considered successful when the complete tumor was within the halo on MRI, since the amount of cell death was proven to correlate well with the MRI aspect [14]. 

Halo volume gradually decreased over time, with about half the volume left after 7 years follow-up. Especially in the first 2 years, this decrease is evident, accounting for an average decrease of 32.6%. This is expected to be the result of remodeling around the halo edge. The reactive zone as seen on the edge of the halo on MRI is hypothesized to correspond with inflamed tissue rather than dead/necrotic tissue. Even though the halo volume decreased over time, no tumor tissue was seen outside the halo edge during follow-up. This advocates for a process of bone revitalization around the edge of the halo. Furthermore, with regard to the fact the average halo volume is already 3.7 times the tumor volume (28.6 vs. 7.67 cm^3^), less aggressive treatment could potentially limit the amount of damaged tissue and therefore lead to a smaller rest defect within less time. However, better predictability of the ablation halo is needed to plan for ablations with a smaller margin. Future research should be focused on visualizing the ablation area to minimize collateral damage. 

Our results are in accordance with literature. Zhao et al. described tissue proliferation and repair to occur between 10 days and 12 weeks after an RFA procedure in six (in vivo) swines, leading to fresh and mature bone trabecula [18]. However, they only evaluated the process for 12 weeks. Bucknor et al. found the volume of the ablation zone to decrease by 35.6% for procedures with high energy and 10.1% for low-energy procedures between 3 and 6 weeks after ultrasound ablation in eight pigs [19]. They reported the regeneration of bone with significantly thicker cortices [20]. Since in the current data volume was first measured after 3 months, it cannot be controlled if a similar halo volume decrease can be found for RFA halos in this period. 

In the current data, there was only one complication and no recurrences were found, meaning that the initial ablation result is a reliable indicator of local tumor control and it is questionable whether long term follow-up is necessary. In contrast, for procedures with incomplete ablation (and subsequently conservative treatment), a profound follow-up still has an important function. Another point of consideration is the fact all volume calculations were carried out based on measurement of MR images instead of histology. However, in the study of Dierselhuis et al., they found MR images to correspond with histological findings [14]. It is expected that new vital tissue develops around the edge of the halo by means of revitalization.

Currently, frequent follow-up (almost annually) is carried out after RFA to control for complications, recurrence, or residual tumor activity. However, the current study indicates that such extensive follow-up is not necessary after complete ablation. For instance, a baseline scan at 3 months followed by a control after 1 year should be sufficient. Apart from reduced treatment intensity, a shorter follow-up might help a patient to finish the experience of being diseased and be able to move on. For incomplete ablations, regular follow-up is still indicated. This hypothesis should be confirmed when our follow-up reaches an average of 7 years to include potential late recurrences.

## 5. Conclusions

Prolonged follow-up demonstrates halo volume to decrease over time indicating bone regeneration. After 7 years, remaining halo volume is only half of its initial value. Ablation result on baseline MRI is a reliable indicator of local tumor control. In 89% of cases, RFA is effective at achieving local tumor control in low-grade cartilaginous tumors of long bone, and complications are scarce.

## Figures and Tables

**Table 1:** General characteristics of the study population.

Variable	Value
Number of patients	82
Gender (M/F)	31/51
Age in years	52.0 (±13.0)
Follow-up time in months	43.8 (19–100)
Treatment result (R0/1/2)	63/10/9
Complications	1
Tumor volume in cm^3^	7.67 (±7.32)
Halo volume baseline in cm^3^	28.6 (±18.1)
Bone (femur/humerus/tibia/fibula)	54/20/7/1
Location (diaphysis/DM/metaphysis) *	20/11/51

* DM: transition diaphysis to metaphysis.

**Table 2:** Proportional decrease in halo dimensions between the follow-up moments. Percentages (and range for percentages) are expressed relatively to baseline. Baseline was separately determined for the samples with a measurement at the specific time interval. Since some patients missed one of the appointments, N is not equal for all follow-up moments.

	*n*	Height	Width	Depth	Volume
Baseline–1 year	73	9.00%	7.65%	8.22%	21.5% (4.55–41.5)
Baseline–2 years	75	13.9%	11.9%	13.3%	33.6% (9.47–69.2)
Baseline–5 years	32	22.5%	18.2%	19.7%	47.8% (15.0–83.6)
Baseline–7 years	7	20.7%	19.1%	19.3%	48.1% (33.4–57.8)

## Data Availability

Data is stored in a database in the university medical center Groningen, the Netherlands and is available upon request.
